# Rare clinical presentation of invasive pulmonary aspergillosis in patient with early-stage lung cancer: a case report

**DOI:** 10.3389/fmed.2026.1894021

**Published:** 2026-09-15

**Authors:** Tatjana Adzić-Vukicevic, Sead Dalifi, Katarina Petkovic, Milan Savic, Katarina Lukic, Dragana Sobic-Saranovic, Jon Salmanton-García

**Affiliations:** 1 Faculty of Medicine, University of Belgrade, Belgrade, Serbia; 2 Clinic for Pulmonology, University Clinical Center of Serbia, Belgrade, Serbia; 3 Clinic for Thoracic Surgery, University Clinical Center of Serbia, Belgrade, Serbia; 4 Center for Radiology and MRI, University Clinical Center of Serbia, Belgrade, Serbia; 5 Center for Nuclear Medicine with PET, University Clinical Center of Serbia, Belgrade, Serbia; 6 Institute of Translational Research, Cologne Excellence Cluster on Cellular Stress Responses in Aging-Associated Diseases (CECAD), Faculty of Medicine and University Hospital Cologne, University of Cologne, Cologne, Germany; 7 Department I of Internal Medicine, Center for Integrated Oncology Aachen Bonn Cologne Duesseldorf (CIO ABCD) and Excellence Center for Medical Mycology (ECMM), Faculty of Medicine and University Hospital Cologne, University of Cologne, Cologne, Germany; 8 German Centre for Infection Research (DZIF), Partner Site Bonn-Cologne, Cologne, Germany

**Keywords:** *Aspergillus niger*, bronchoscopy, invasive pulmonary aspergillosis, lung cancer, voriconazole

## Abstract

Invasive pulmonary aspergillosis (IPA) presents life-threatening complication mostly seen in patients with severe neutropenia or in patients with solid organ cancers after applied anticancer therapy. The aim of this manuscript is to identify clinical manifestations of IPA in lung cancer patient. A 65-year-old male with a history of severe chronic obstructive lung disease and prior laryngeal carcinoma was admitted to hospital due to fever. Five months before he was operated because of early-stage squamous cell lung carcinoma. Postoperative course was complicated with subcutaneous emphysema and recurrent pneumothorax. The appearance of new excavated lesions in the contralateral lobes on repeated chest computed tomography (CT) raised concern for tumor recurrence. Subsequent bronchoscopy with bronchoaspirate culture yielded *Aspergillus niger*. Molecular tests also showed positive findings for *Aspergillus* presence. First-line treatment with voriconazole resulted in significant clinical and radiological improvement. Positron emission tomography (PET-CT) done 6 months after voriconazole treatment showed only mild fluorodeoxyglucose (FDG) uptake in the right lower lobe, with no other areas of involvement This case highlights the importance of a multidisciplinary approach in lung cancer patients, where post-operative radiologic changes can represent tumor recurrence, bacterial infection or fungal disease.

## Introduction

Aspergillosis includes various diseases caused by *Aspergillus* species dependent upon the host immunological responses. *Aspergillus* is a ubiquitous filamentous fungus, commonly found in soil, water, and decaying organic matter, whose spores can be inhaled and, under favorable conditions, invade lung tissue (1).

Disease manifestations vary according to host immunity: in immunocompromised patients, angioinvasion, tissue necrosis, and hemorrhagic infarction are typical, whereas in patients with pre-existing lung conditions, such as chronic obstructive pulmonary disease (COPD), subacute or chronic forms can occur without angioinvasion (2). Allergic bronchopulmonary aspergillosis (ABPA) presents an immune-mediated response to fungal antigens, while aspergilloma and chronic pulmonary aspergillosis develop in pre-existing lung cavities without tissue invasion (3).

Invasive pulmonary aspergillosis (IPA) is a severe fungal infection primarily caused by *Aspergillus* species, associated with high morbidity and mortality, particularly in immunocompromised patients (3, 4). The most commonly implicated pathogens in IPA are the following: *Aspergillus fumigatus*, *Aspergillus flavus*, *Aspergillus terreus* and *Aspergillus niger*. *Aspergillus fumigatus* is the most frequently reported accounting for 60–90% of all cases. *A. niger* is generally low in virulence and rarely causes invasive disease. Its larger size (spores measure between 3.5 μm to 5 μm in diameter) compared to other *Aspergillus* species, limits its dissemination to the lower respiratory tract (4). It has been reported that in patients with COPD and IPA, only 3.6% of isolates revealed presence of *A. niger* (5).

Classical risk factors for iPA include prolonged neutropenia, cytotoxic chemotherapy, and extended corticosteroid therapy, with reported mortality rates reaching up to 60% (3). While hematologic malignancies and transplant recipients are at highest risk, IPA has been increasingly reported among solid tumor patients, particularly those with structural lung disease, prior irradiation or prolonged corticosteroid therapy (6, 7).

Lung cancer is the most common malignancy worldwide and the leading cause of cancer-related death, with an estimated 2.48 million new cases and 1.8 million deaths annually (8). The overall five-year survival for non-small cell lung cancer remains approximately 20%. Due to changes in respiratory structure, lung cancer patients are more susceptible to the growth of pathogenic microorganisms. Bronchial colonization also plays an important role in lung infections. Lung cancer patients are more prone to *Aspergillus* colonization and IPA up to 25.8%. In inoperable lung cancer patients, tumor infiltration into the bone marrow and chemoradiotherapy often lead to immunosuppression with lymphocyte reduction and increase risk for infections and death. Therefore, in operated lung cancer patients, postoperative follow-up should be addressed to infectious complications, not only on tumor recurrence (9). Postoperative patients may experience transient immunosuppression, altered pulmonary architecture, or prolonged intubation, which can increase susceptibility to fungal invasion. Novel therapies, including targeted agents such as gefitinib or bevacizumab, and immune checkpoint inhibitors, may further modulate host immunity, indirectly increasing IPA risk (10–13).

The diagnosis of IPA is challenging due to nonspecific clinical features, including fever, cough, dyspnea, and hemoptysis, which often overlap with bacterial infections or tumor recurrence. Imaging studies play a central role: angio-invasive forms typically present on CT as nodules with surrounding ground-glass opacities (“halo sign”), whereas airway-invasive patterns produce centrilobular nodules with a *tree-in-bud* appearance (14). Bronchoscopy with bronchoalveolar lavage, microbiologic cultures, molecular assays [galactomannan, 1,3-β-D-glucan, polymerase chain reaction (PCR)], and histopathology remain essential for accurate diagnosis (15). Positron emission tomography computed tomography (PET-CT) may assist in differentiating active infection from post-surgical changes or malignancy (16).

Treatment of IPA relies on azole antifungals, primarily voriconazole, with amphotericin B or echinocandins reserved for refractory cases. Therapy is prolonged and requires monitoring for adverse effects, including hepatotoxicity, photosensitivity, and, rarely, cardiac arrhythmias. Surgical intervention may be considered in cases unresponsive to medical therapy, guided by pulmonary function assessment (15, 16).

This report describes a rare case of IPA following radical lung cancer resection, emphasizing the need for heightened clinical awareness in postoperative patients, even in the absence of classical immunosuppressive conditions. Recognition of IPA in this setting is critical to initiate timely antifungal therapy and improve clinical outcomes.

## Case description

A 65-year-old male, a former smoker with a 25-year smoking history, was admitted to the Pulmonology Clinic of the University Clinical Centre of Serbia, Belgrade, with fever up to 39 °C, productive cough, and general weakness. His medical history included laryngeal carcinoma treated with radiation therapy 8 years earlier.

Five months before the current admission, the patient had been hospitalized in the Thoracic Surgery Clinic of the University Clinical Centre of Serbia, Belgrade, for a partial left-sided pneumothorax (Figure 1A), which was managed with thoracic drainage. After complete lung re-expansion, a thoracic CT scan revealed an excavated lesion in the left upper lobe, along with bullous changes in both lungs (Figure 1B). Pulmonary function tests showed severe obstructive lung disease, with forced vital capacity (FVC) 77% predicted (3.66 L), forced expiratory volume in the first second (FEV_1_) 36% predicted (1.31 L), FEV_1_/FVC ratio 35.8%, transfer factor for carbon monoxide (TLCO) 55% predicted, and carbon monoxide transfer coefficient (KCO) 58% predicted.

**Figure 1:**
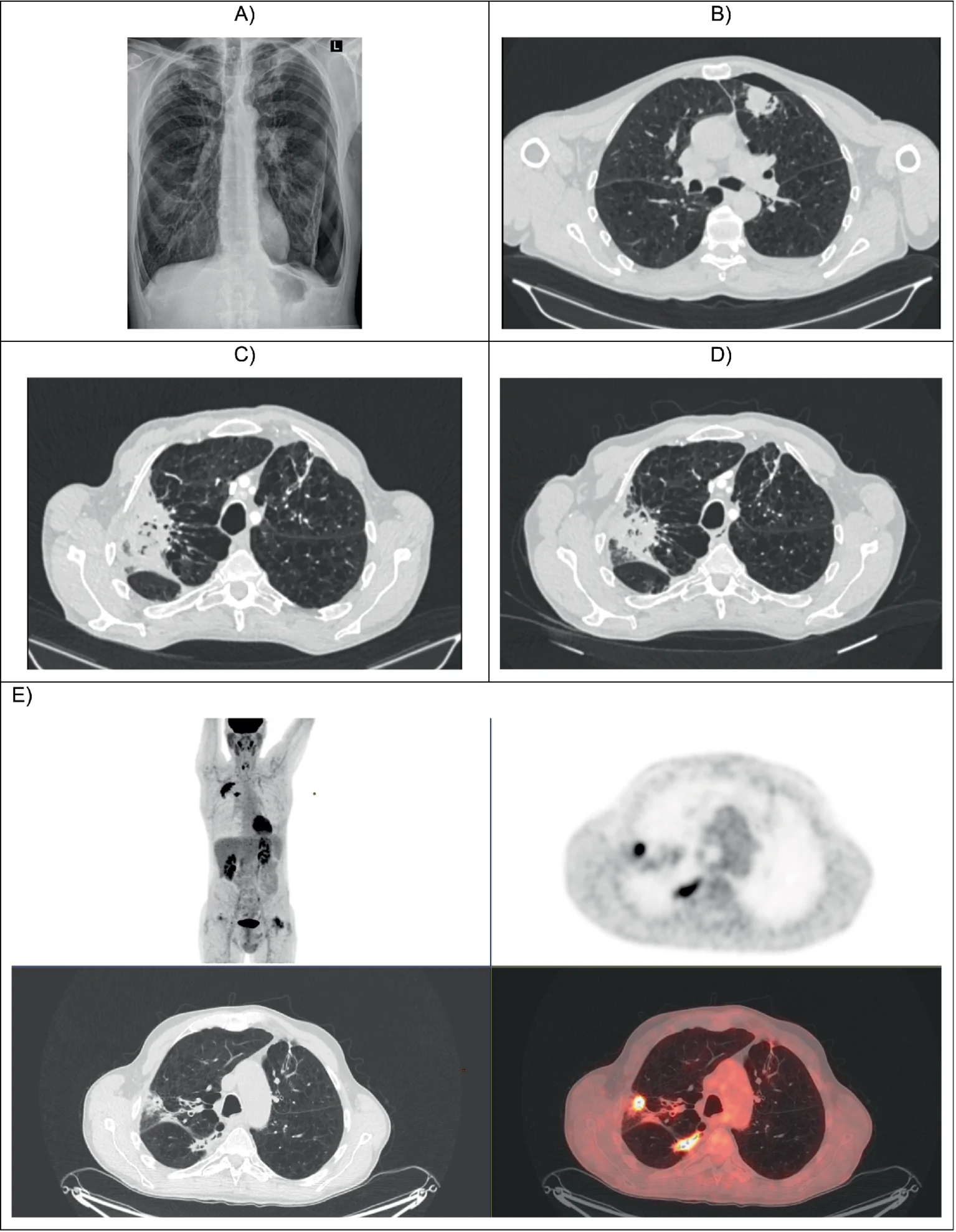
Thorax radiography, computed tomography and PET CT findings. **(A)** Partial pneumothorax of the left lung with radiological signs of hyperinflation; **(B)** Cavitary lesion in the left upper lobe with a small adjacent pneumothorax; **(C)** Cavitary lesion in the posterior segment of the right upper lobe with an additional lesion in the superior segment of the right lower lobe; **(D)** Regression of the right-sided cavitary lesions after 6 months of voriconazole therapy; **(E)** PET-CT following 6 months of voriconazole therapy showed mild fluorodeoxyglucose uptake in the right lower lobe with no other areas of involvement.

After intensive deobstructive therapy, the patient underwent video-assisted thoracic surgery (VATS) with atypical resection of the left upper lobe. Histopathology confirmed squamous cell lung carcinoma, staged as T1cN1R0PL0, with a tumor size of 30 × 30 × 28 mm. Immunohistochemistry showed programmed death-ligand 1 (PD-L1) expression of 10% (Figure 2).

**Figure 2:**
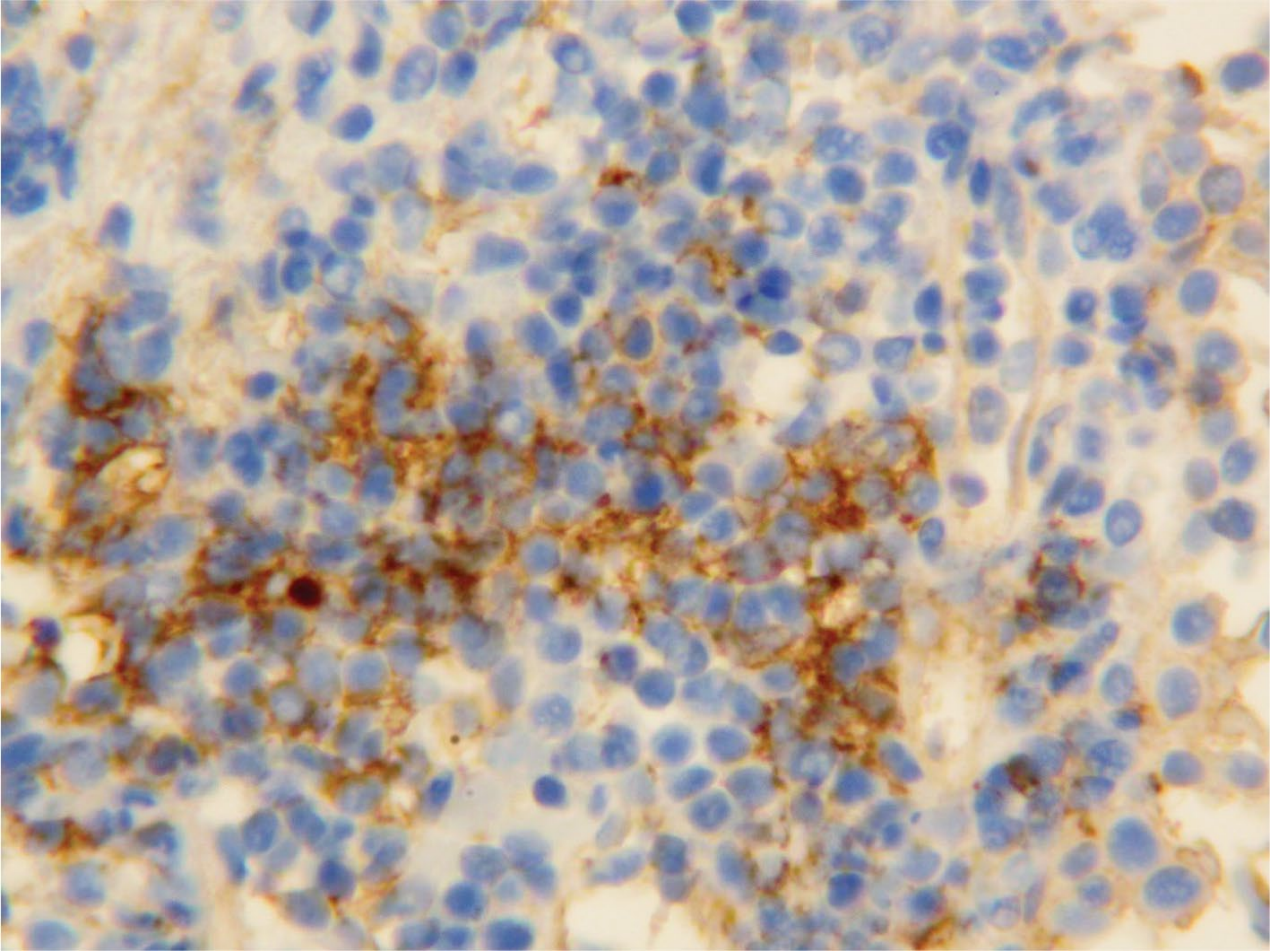
Immunohistochemical analysis of lung cancer biopsy (enlargement x40). Immunohistochemical analysis shows a low differentiated lung squamous cell carcinoma with PD-L1 expression detected in 10% of tumor cells. Tumor cells are also surrounded by a few positive stromal lymphocytes.

The postoperative course was complicated by subcutaneous emphysema and recurrent left-sided pneumothorax, requiring a second drainage and subsequent management with a Heimlich valve system. Six weeks later, the valve was removed, and the lung had fully re-expanded. During this period, the patient received multiple courses of antibiotics like second and third line cephalosporins (ceftriaxone 2 gr/day/7 days than ceftazidime 3×2 gr/day/7 days), fluoroquinolones (moxifloxacin 400 mg/day/10 days) and metronidazole (3×400 mg/day/10 days). After recovery, three cycles of adjuvant chemotherapy with gemcitabine (G) and platinum (P) were planned. However, the first cycle was not completed due to fever during the scheduled mono-gemcitabine treatment on day eight.

A new excavated lesion in the right upper lobe was found on chest X ray. Laboratory tests showed elevated C-reactive protein (CRP, 198 mg/L; normal <5 mg/L) and leukocytosis (white blood cell count 20.3 × 10^9^/L) with neutrophil predominance (15.5%). Sputum cultures were negative, and Ziehl-Neelsen staining showed no evidence of *Mycobacterium tuberculosis*. Empirical intravenous antibiotics (ceftriaxone 2 g/day and levofloxacin 500 mg twice daily) were administered without improvement.

A thoracic CT scan revealed a 43 × 32 mm excavated lesion in the posterior segment of the right upper lobe and another lesion (41 × 18 mm) in the superior segment of the right lower lobe (Figure 1C). These changes were absent on previous CT scans. Due to concern for tumor recurrence, fiberoptic bronchoscopy with fine-needle aspiration of the right lung cavity was performed. Forceps biopsy of bronchial tissue showed only inflammatory cells, without malignancy. Samples from bronchoaspirate were added to Sabouraud dextrose agar (Sabouraud dextrose/maltose agar, Torlak, Serbia), on 28 °C for 2–6 days. The fungi were first identified on the basis of light microscopis features (septate branching hyphae and long conidiophores terminating in large spherical vesicles). Protein analysis was performed using a matrix-assisted laser desorption ionization time on flight mass spectrometry (MALDITOF-MS) for *A. niger* identification. Follow-up testing revealed elevated 1,3-β-D-glucan (BDG) (9.025; cut-off <120) and a positive quantitative polymerase chain reaction (PCR) for *Aspergillus* deoxyribonucleic acid (DNA) (1,216 copies/mL; positive ≥120) (ELITe MGB Kit; ELITechGroup S.p.A, Italy).

The patient met the criteria for IPA, and antifungal therapy with intravenous voriconazole was initiated (intravenous 6 mg/kg twice daily for the first day, continued with 4 mg/kg twice daily for 14 days), later switched to oral therapy (200 mg twice daily) for 6 months. The treatment was well tolerated except for a brief episode of ventricular arrhythmias, which resolved with beta-blockers (bisoprolol 2.5 mg/day).

Six months later, CT showed regression of the right upper lobe lesion, with a persistent cavity in the right lower lobe (Figure 1D). Follow-up bronchoscopy with transbronchial needle aspiration was negative for malignancy, and bronchoaspirate testing was negative for *Aspergillus* on culture, PCR, and 1,3-β-D-glucan. PET-CT showed only mild fluorodeoxyglucose (FDG) uptake in the right lower lobe, with no other areas of involvement (Figure 1E). Voriconazole treatment was continued, and the patient’s general condition improved. Adjuvant chemotherapy was not continued because of prolonged period after first cycle of chemotherapy and period without lung cancer recurrence. Figure 3 shows clinical course of disease.

**Figure 3:**
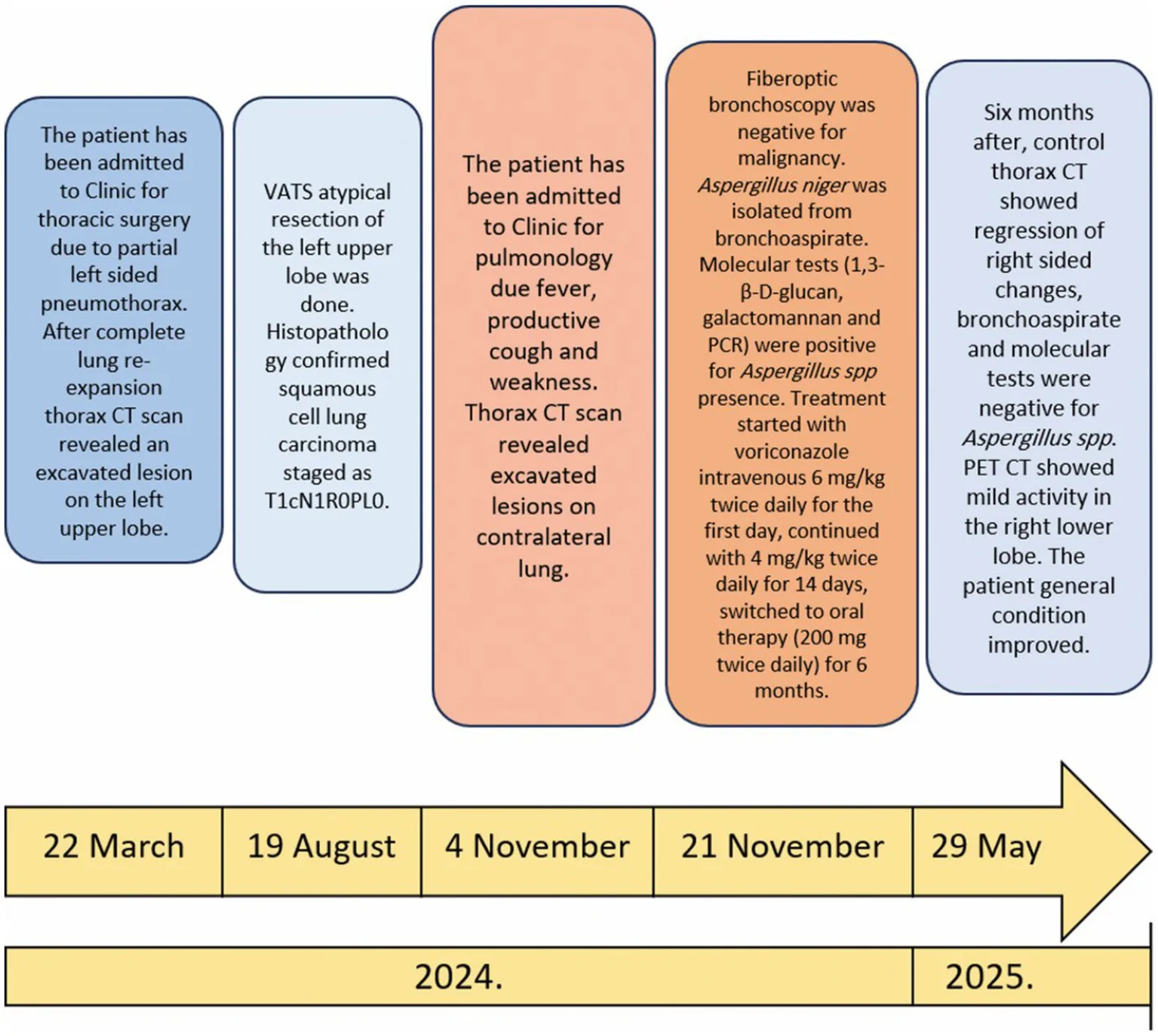
Timeline of clinical course.

## Discussion

Our patient was a 65-year-old male with a history of COPD and severe obstructive lung disease, prior laryngeal carcinoma treated with radiation therapy and a significant smoking history. Five months before the current admission, he underwent VATS with atypical resection of the left upper lobe for early-stage squamous cell lung carcinoma. Postoperative complications included subcutaneous emphysema and recurrent pneumothorax, requiring prolonged drainage with a Heimlich valve. These factors, combined with impaired pulmonary function (TLCO 55%, FEV_1_ 36% predicted), placed him at increased risk for pulmonary infections, including IPA.

The differential diagnosis in this patient was complex. The appearance of new excavated lesions in the right upper and lower lobes raised concern for tumor recurrence or bacterial infection. Laboratory work-up revealed leukocytosis with neutrophil predominance and markedly elevated CRP (198 mg/L), while sputum cultures and Ziehl–Neelsen staining were negative. Notably, empirical broad-spectrum antibiotics failed to produce clinical or radiological improvement. Subsequent bronchoscopy with bronchoaspirate culture yielded *A. niger*, and molecular testing confirmed infection with elevated 1,3-β-D-glucan and a positive quantitative PCR for *Aspergillus* DNA, satisfying criteria for IPA diagnosis. *A. niger* is less commonly associated with IPA than other species of *Aspergillus* usually associated with cutaneous infections and otomycosis (1, 2).

Structural lung disease, such as COPD, is a recognized as predisposing factor for IPA, even in the absence of profound neutropenia. In this patient, severe obstructive lung disease and post-surgical remodeling likely facilitated fungal colonization and subsequent invasive disease (3). Furthermore, chemotherapy planned as adjuvant GP therapy could have further impaired immune defenses, although treatment was not completed due to febrile episodes. While IPA is rare in early-stage lung cancer patients, prior studies have reported an incidence of approximately 2.6% (8). This highlights the importance of considering IPA in the differential diagnosis of post-operative pulmonary lesions, especially when empirical antibiotic therapy fails.

Radiological evaluation plays a crucial role in the diagnosis and monitoring of disease progression. Two different types of IPA could be described on CT scans. The first one include angio-invasive pattern happened by hyphae penetration through blood vessels wall leading to fungal thrombi causing necrosis and hematogenous dissemination. This CT finding is usually presented with nodules surrounded by ground-glass opacities, perilesional hemorrhagic with” halo sign” formation. The” halo sign” strongly suggest IPA in severe neutropenic patients but it can be seen in other conditions like primary or metastatic cancer, bacterial infections, granulomatous diseases and bronchiolitis obliterans with organizing pneumonia. Other type of CT finding in IPA is airway-invasive pattern which is mostly seen in non neutropenic patient due to endobronchial dissemination of fungal infection with multiple centrilobular nodules resulting in the typical” tree-in-bud” pattern. This finding can also be found in various pulmonary infections, from mycobacteria to viruses (14, 15). Our patients' thorax CT revealed excavated lesions without the classic angio-invasive” halo sign” typical of neutropenic patients, consistent with airway-invasive IPA patterns seen in non-neutropenic hosts. The analysis of fluorodeoxyglucose (FDG) uptake is useful for better evaluation and extent of lesions (16). PET-CT further aided in differentiating active infection from residual post-surgical changes, guiding the continuation of antifungal therapy (16).

First-line treatment with voriconazole resulted in significant clinical and radiological improvement, with 6 months of therapy leading to regression of the right upper lobe lesion and persistent but inactive cavity in the right lower lobe. Notably, the patient experienced ventricular extrasystoles during treatment, a rare but recognized cardiac adverse effect of voriconazole due to inhibition of potassium channels in cardiac myocytes and potential QT prolongation. These were successfully managed with beta-blockers. The patient tolerated long-term oral therapy well, with improvement in general condition and weight gain of 8 kilograms.

Surgical resection for IPA is generally recommended for patients with localized disease unresponsive to antifungal therapy or in cases of aspergilloma. However, in our patient, poor lung function (TLCO <40% predicted) contraindicated right upper lobectomy due to high postoperative morbidity and mortality. This case illustrates the potential for medical management alone to achieve favorable outcomes in high-risk surgical candidates Multidisciplinary approach in lung cancer patients is mandatory, especially when post-operative radiologic changes can represent tumor recurrence, bacterial infection or fungal disease. Accurate diagnosis requires integration of imaging, microbiology, immunology, molecular diagnostics and histopathology. In particular, bronchoscopy with bronchoalveolar lavage cultures and molecular tests such as PCR and 1,3-β-D-glucan are essential when sputum cultures are negative, as demonstrated in this case. Voriconazole can lead to substantial clinical improvement even in patients with high surgical risk, although careful monitoring for rare cardiac adverse events is warranted (17). Cardiac arrhythmias are unusual and rare complication of voriconazole use. Potentially life-threatening arrhythmias with prolonged and dispersed QT interval were described in patients receiving intravenous voriconazole (18). Baseline electrocardiograph screening should be performed before voriconazole administration and during the treatment primarily based on patients age and comorbidities. Our case has a few limitations, monitoring of serum voriconazole level was not done as well as PET-CT at the beginning of voriconazole treatment.

In conclusions, IPA can express atypical imaging characteristics in patients with early-stage lung cancer, however it should be considered in the differential diagnosis of post-operative pulmonary lesions to avoid misdiagnosis as tumor recurrence. Chest computed tomography plays a crucial role in the diagnosis of IPA confirming excavated lesions without the classic angio-invasive” halo” typical of neutropenic patients. Finally, rare species of *Aspergillus* like *A. niger* can cause IPA in patients with early-stage lung cancer.

## Data Availability

The data supporting the findings of this case report are not publicly available due to patient privacy and confidentiality restrictions. Requests to access the datasets should be directed to sead.dalifi@yahoo.com.
